# Triglyceride–Glucose Index and Short‐Term Functional Prognosis in Patients With Acute Ischemic Stroke: A Retrospective Study

**DOI:** 10.1002/brb3.71013

**Published:** 2025-10-29

**Authors:** Zhuqing Wu, Juan Shi, Yueyu Zhang, Chi Zhang, Qiuwan Liu, Xiaoqiang Wang, Kangrui Zhang, Juncang Wu

**Affiliations:** ^1^ Department of Neurology The Second People's Hospital of Hefei, Hefei Hospital Affiliated of Anhui Medical University Hefei Anhui China; ^2^ Department of Operating Room The Second Affiliated Hospital of Anhui Medical University Hefei Anhui China

**Keywords:** acute ischemic stroke, insulin resistance, prognosis, triglyceride–glucose index, TyG‐NIHSS

## Abstract

**Background:**

Stroke is the most significant cause of death and disability around the world. It is the second leading cause of death after cardiovascular disease. Currently, the triglyceride–glucose (TyG) index has proven to be a reliable surrogate indicator of IR in stroke studies. However, the relationship between TyG and poor functional outcomes in patients with ischemic stroke remains unclear. Accordingly, this study aimed to explore the relationship between TyG index and clinical outcomes at 3 months after acute ischemic stroke (AIS).

**Methods:**

The clinical data of 564 AIS patients admitted to the Second People's Hospital of Hefei from January 2020 to September 2024 were collected. According to the mRS score at 3 months after onset, the patients were divided into a poor functional prognosis group and a good functional prognosis group. Univariate and multivariate logistic regression models were used to explore the correlation between the TyG index at admission and the 3‐month functional prognosis of AIS patients. The receiver operating characteristic (ROC) curve was used to evaluate the predictive ability of the TyG index and the TyG index combined with the admission NIHSS score (TyG‐NIHSS) for the 3‐month functional prognosis of AIS patients.

**Results:**

A total of 564 AIS patients were included, with 165 cases (29.25%) in the poor functional prognosis group and 399 cases (70.75%) in the good functional prognosis group. Multivariate logistic regression analysis showed that systolic blood pressure at admission, NIHSS score, and TyG index were independent risk factors for poor functional prognosis at 3 months in AIS patients (*p* < 0.05). The higher the TyG index, the higher the risk of poor functional prognosis at 3 months (OR = 3.18, 95% CI: 2.252–4.499, *p* < 0.001). ROC curve analysis showed that the area under the curve (AUC) for the TyG index to predict poor functional prognosis at 3 months in AIS patients was 0.650 (95% CI: 0.598–0.702, *p* < 0.001), with a sensitivity of 61.2% and a specificity of 62.7%. The AUC for TyG‐NIHSS to predict poor functional prognosis at 3 months in AIS patients was 0.836 (95% CI: 0.799–0.873, *p *< 0.001), with a sensitivity of 80.6% and a specificity of 76.7%.

**Conclusion:**

The TyG index is an independent but moderate predictor of poor outcomes at 3 months poststroke. However, TyG‐NIHSS represents a highly discriminative multivariate model. This model demonstrates good predictive ability and high predictive accuracy.

## Graphical abstract

A high triglyceride–glucose (TyG) index is an independent risk factor for poor functional outcome at 3 months in patients with acute ischemic stroke (AIS). The predictive accuracy of the combined TyG‐NIHSS model for a poor 3‐month outcome was significantly higher than that of the TyG index alone

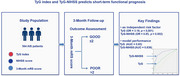

## 1 Introduction

Stroke is currently the leading cause of death and disability worldwide, and the second leading cause of death from cardiovascular disease (Vos et al. 2020). In China, stroke is the leading cause of death and disability among residents (Wu et al. 2019). Acute ischemic stroke (AIS) is the most common type of stroke, accounting for about 72.8% of new strokes, with a 1‐year mortality and disability rate of 6% and 14.2% (Wang et al. 2022; Tu et al. 2021). Therefore, it is particularly important to accurately assess the prognosis of AIS patients in order to guide early risk stratification and improve treatment, thereby reducing the disability and mortality rates of stroke. It is well known that insulin resistance (IR) is a risk factor for stroke (Kernan et al. 2002; Brie et al. 2025). By promoting atherosclerosis (Yang et al. 2023), inducing hemodynamic disturbances (Cao et al. 2024) and activating platelets (Kelem et al. 2023), it participates in the occurrence, development, and prognosis of stroke. In clinical practice, IR requires the measurement of insulin and other indicators, which are expensive and not easily available in clinical practice, and their application is greatly limited. The triglyceride–glucose (TyG) index is a biological marker of fasting triglycerides and glucose levels, which can be used to screen and predict various diseases, especially those related to IR and metabolic syndrome. It has been confirmed that the TyG index can be used as a reliable alternative indicator for IR (Li et al. 2023). It was first used for cardiovascular diseases, and its correlation with stroke is still relatively limited, mostly focusing on the incidence and recurrence of stroke, and there are relatively few studies on the correlation with prognosis (Nayak et al. 2024). In order to better explore the correlation between the TyG index and the prognosis of AIS patients, we conducted this study on a population in our center to investigate the relationship between the TyG index and the early functional prognosis of AIS patients.

## 2 Methods

### 2.1 Study Design and Setting

The retrospective study was conducted at the National Advanced Stroke Center of the Second People's Hospital within the Hefei, Anhui Province, China. All of the data were collected retrospectively through the central electronic medical records system. A total of 564 patients who met predetermined inclusion criteria were enrolled in the study. Their medical records were reviewed and recorded by a neurologist. The study was conducted over a 3‐month period, during which patient data were collected and analyzed to meet the study's objectives.

### 2.2 Study Population

The study subjects were AIS patients admitted to the advanced stroke center of the Second People's Hospital of Hefei from January 2020 to September 2024. The inclusion criteria were: (1) age ≥ 18 years and (2) diagnosis of AIS according to the diagnostic criteria established by the World Health Organization, based on patient history, clinical data, and neurological imaging (including diffusion‐weighted imaging). The exclusion criteria were: (1) admission time > 7 days from onset and (2) lack of fasting blood glucose and triglyceride data. Finally, 564 patients with AIS were included in this study.

### 2.3 Triglyceride–Glucose Index

Fasting venous blood samples were collected within 24 h of admission, and fasting triglyceride and glucose were measured by enzymatic method. The TyG index was calculated as ln [fasting triglycerides (mg/dL) × fasting glucose (mg/dL) / 2] (Guerrero‐Romero et al. 2010). The necessary data for this calculation were obtained from patient case reports, laboratory test results, and the completed data collection forms.

### 2.4 Data Collection

Data on patients' demographic characteristics, medical history, clinical characteristics, and laboratory tests were collected from the hospital's electronic medical record system. This included age, sex, body mass index (BMI), hypertension, diabetes, coronary heart disease, history of stroke, atrial fibrillation, smoking history, drinking history, systolic blood pressure at admission, diastolic blood pressure at admission, and biochemical indicators, as well as NIHSS and mRS scores at admission and 3 months after onset.

### 2.5 Prognosis Evaluation and Grouping

The severity of stroke and prognosis were evaluated using the National Institute of Health Stroke Scale (NIHSS) and the modified Rankin Scale (mRS). The mRS is a scale for assessing the degree of disability or dependence in daily activities of patients with stroke or other neurological diseases (Broderick et al. 2017). The mRS is graded 0–6 to assess the extent of functional impairment. The grades are: 0: no symptoms; no disability, able to perform all previous activities (Score 0). I: mild disability; unable to perform all previous activities, but able to handle personal affairs without assistance (Score 1). II: mild disability; unable to handle personal affairs without assistance (Score 2). III: moderate disability; requiring some help, but able to walk independently (Score 3). IV: moderately severe disability; unable to walk independently and requiring assistance with daily activities (Score 4). V: severe disability; bedridden, incontinent, requiring constant nursing care (Score 5). VI: death (Score 6) (de Havenon et al. 2021).

Patients were divided into a good functional prognosis group and a poor functional prognosis group. Good functional prognosis was defined as an mRS score of ≤ 2; poor functional prognosis was defined as an mRS score of 3–6.

### 2.6 Statistical Analysis

SPSS 22.0 was used for statistical description and analysis of the collected data. Normally distributed measurement data were described by mean ± standard deviation (*x* ± *s*), and non‐normally distributed data were described by median (interquartile range) (*M*, IQR). Count data were described by frequency and percentage (*n*, %). The comparison between two groups of non‐normally distributed data was performed by Mann–Whitney *U* test, and the comparison of count data was performed by the *t*‐test or *χ* ^2^ test or Fisher's exact test. Univariate and multivariate logistic regression models were used to explore the correlation between the TyG index at admission and the early functional prognosis of AIS patients. The receiver operating characteristic (ROC) curve was used to evaluate the predictive value of the TyG index for the 3‐month functional prognosis of AIS patients. The optimal cut‐off value of the TyG index for predicting poor functional outcomes was determined using the maximum Youden index method, which achieved the optimal balance between sensitivity and specificity. All statistical test levels in this study were defined as bilateral test *p* < 0.05.

## 3 Results

### 3.1 Baseline Characteristics

According to the inclusion and exclusion criteria, a total of 564 AIS patients were included, with 165 cases (29.25%) in the poor functional prognosis group and 399 cases (70.75%) in the good functional prognosis group. The clinical data and laboratory examinations of the two groups were compared. Among them, hypertension, diabetes, atrial fibrillation, systolic blood pressure at admission, NIHSS score at admission, fasting blood glucose, triglycerides, HDL, and TyG index were statistically significant between the two groups (*p* < 0.05) (Table 1).

**TABLE 1:** Baseline characteristics and 3‐month functional prognosis.

Variables	Poor prognosis group (*n* = 165)	Good prognosis group (*n* = 399)	*p*‐value
**Demography**			
Age (years, mean ± SD)	66.20 ± 11.612	65.13 ± 11.351	0.310
Male (*n*, %)	103 (62.42%)	278 (69.67%)	0.094
BMI (kg/m^2^, mean ± SD)	24.45 ± 3.641	24.32 ± 3.231	0.689
**Personal history (*n*, %)**			
Smoking	46 (27.88%)	128 (32.08%)	0.326
Alcohol	37 (22.42%)	115 (28.82%)	0.119
**Medical history (*n*, %)**			
Hypertension	127 (76.97%)	274 (68.67%)	0.048
DM	77 (46.67%)	121 (30.32%)	0.000
HLP	14 (8.48%)	24 (6.01%)	0.287
CHD	19 (11.51%)	27 (6.77%)	0.061
AF	18 (10.91%)	21 (5.26%)	0.016
Stroke	61 (36.97%)	132 (33.08%)	0.376
**Clinical features of admission**			
SBP (mmHg, mean ± SD)	154.68 ± 21.021	149.69 ± 19.926	0.008
DBP (mmHg, mean ± SD)	85.53 ± 12.612	84.71 ± 13.532	0.504
NIHSS (*M*, IQR)	7 (5, 11)	3 (2, 5)	0.000
Thrombolytic therapy (*n*, %)	66 (40.00%)	97 (24.31%)	0.000
Laboratory inspection			
FBG (mmol/L, *M*, IQR)	6.91 (5.59, 10.22)	5.60 (4.90, 7.07)	0.000
BUN (µmol/L, *M*, IQR)	5.37 (4.31, 6.53)	5.14 (4.30, 6.50)	0.641
CRE (µmol/L, mean ± SD)	70.75 ± 28.471	67.76 ± 21.212	0.225
UA (µmol/L, mean ± SD)	330.14 ± 99.255	329.20 ± 83.782	0.915
TG (mmol/L, *M*, IQR)	1.56 (1.10, 2.19)	1.29 (0.96, 1.77)	0.000
TCHO (mmol/L, mean ± SD)	4.56 ± 1.267	4.55 ± 1.582	0.914
HDL (mmol/L, *M*, IQR)	1.13 (0.94, 1.30)	1.17 (0.99, 1.37)	0.049
LDL (mmol/L, mean ± SD)	2.89 ± 0.950	2.88 ± 0.967	0.991
TyG index (*M*, IQR)	9.05 (8.60, 9.70)	8.73 (8.37, 9.14)	0.000

*Note*: Data are the mean ± standard deviation, number (percentage), or median (interquartile range).

Abbreviations: AF, atrial fibrillation; BMI, body mass index; BUN, blood urea nitrogen; CHD, coronary heart disease; CRE, creatinine; DBP, diastolic blood pressure; DM, diabetes mellitus; FBG, fasting blood glucose; HDL, high‐density lipoprotein; HLP, hyperlipidemia; LDL, low‐density lipoprotein; NIHSS, National Institutes of Health Stroke Scale; SBP, systolic blood pressure; TCHO, total cholesterol; TG, triglyceride; TyG index, triglyceride–glucose index; UA, uric acid.

### 3.2 Risk Factors for 3‐Month Poor Functional Prognosis

Univariate logistic regression analysis showed that a history of hypertension, diabetes, atrial fibrillation, systolic blood pressure at admission, NIHSS score at admission, fasting blood glucose, triglycerides, and TyG index at admission were all related to poor functional prognosis at 3 months, and the differences were statistically significant (*p* < 0.05) (Table 2).

**TABLE 2:** Risk factors for 3‐month poor functional prognosis.

Variables	B	S.E.	Wals	HR	95% CI	*p*‐value
Age	0.008	0.008	1.077	1.009	0.993–1.025	0.299
Male	−0.312	0.194	2.593	0.732	0.500–1.070	0.107
BMI	0.011	0.028	0.162	1.011	0.958–1.067	0.687
Smoking	0.189	0.204	0.856	1.208	0.810–1.802	0.355
Alcohol	0.337	0.217	2.414	1.401	0.916–2.143	0.120
Hypertension	−0.422	0.214	3.881	0.656	0.431–0.998	0.049
DM	−0.686	0.190	13.033	0.503	0.347–0.731	0.000
HLP	−0.327	0.347	0.887	0.721	0.365–1.425	0.346
CHD	−0.584	0.315	3.437	0.558	0.301–1.034	0.064
AF	−0.790	0.336	5.546	0.454	0.235–0.876	0.019
Stroke	−0.171	0.193	0.783	0.843	0.577–1.231	0.376
SBP of admission	0.012	0.005	7.169	1.012	1.003–1.022	0.007
DBP of admission	0.005	0.007	0.467	1.005	0.991–1.019	0.494
NIHSS of admission	0.229	0.026	78.898	1.257	1.195–1.322	0.000
Thrombolytic therapy	−0.731	0.197	13.718	0.482	0.327–0.709	0.000
FBG	0.210	0.033	41.663	1.234	1.157–1.315	0.000
BUN	−0.011	0.012	0.876	0.989	0.966–1.012	0.349
CRE	0.005	0.004	1.869	1.005	0.998–1.013	0.172
UA	0.000	0.001	0.010	1.000	0.998–1.002	0.922
TG	0.296	0.082	12.917	1.344	1.144–1.579	0.000
TCHO	0.005	0.062	0.006	1.005	0.890–1.134	0.939
HDL	−0.027	0.074	0.137	0.973	0.842–1.124	0.711
LDL	−0.001	0.097	0.000	0.999	0.827–1.207	0.992
TyG index	0.881	0.141	39.140	2.412	1.831–3.179	0.000

Abbreviations: AF, atrial fibrillation; BMI, body mass index; BUN, blood urea nitrogen; CHD, coronary heart disease; CRE, creatinine; DBP, diastolic blood pressure; DM, diabetes mellitus; FBG, fasting blood glucose; HDL, high‐density lipoprotein; HLP, hyperlipidemia; LDL, low‐density lipoprotein; NIHSS, National Institutes of Health Stroke Scale; SBP, systolic blood pressure; TCHO, total cholesterol; TG, triglyceride; TyG index, triglyceride–glucose index; UA, uric acid.

### 3.3 Independent Risk Factors for 3‐Month Poor Functional Prognosis

Multivariate logistic regression analysis was performed with hypertension, diabetes, atrial fibrillation, systolic blood pressure at admission, NIHSS score at admission, fasting blood glucose, triglycerides, and TyG index as independent variables. The results showed that systolic blood pressure at admission, NIHSS score at admission, and TyG index were independent risk factors for poor functional prognosis at 3 months in AIS patients (*p* < 0.05). The higher the TyG index at admission, the higher the risk of poor functional prognosis at 3 months (OR = 3.18, 95% CI = 2.252–4.499, *p* < 0.001) (Table 3).

**TABLE 3:** Independent risk factors for 3‐month poor functional prognosis.

Variables	B	S.E.	Wals	HR	95% CI	*p*‐value
Hypertension	0.162	0.273	0.352	1.176	0.688–2.009	0.553
DM	0.411	0.254	2.627	1.508	0.918–2.479	0.105
AF	0.061	0.489	0.015	1.063	0.407–2.773	0.901
SBP of admission	−0.011	0.006	4.025	0.989	0.978–1.000	0.045
NIHSS of admission	1.109	0.441	6.320	3.033	1.277–7.203	0.012
Thrombolytic therapy	0.242	0.278	0.756	0.785	0.456–1.354	0.384
TyG index	−1.158	0.177	42.983	3.183	2.252–4.499	0.000
TyG‐NIHSS	−0.163	0.052	10.014	0.850	0.768–0.940	0.002

Abbreviations: AF, atrial fibrillation; DM, diabetes mellitus; NIHSS, National Institutes of Health Stroke Scale; SBP, systolic blood pressure; TyG index, triglyceride–glucose index; TyG‐NIHSS, The triglyceride–glucose index combined with the admission NIHSS score.

### 3.4 Predictive Ability of Independent Risk Factors for 3‐Month Poor Functional Prognosis

**FIGURE 1:**
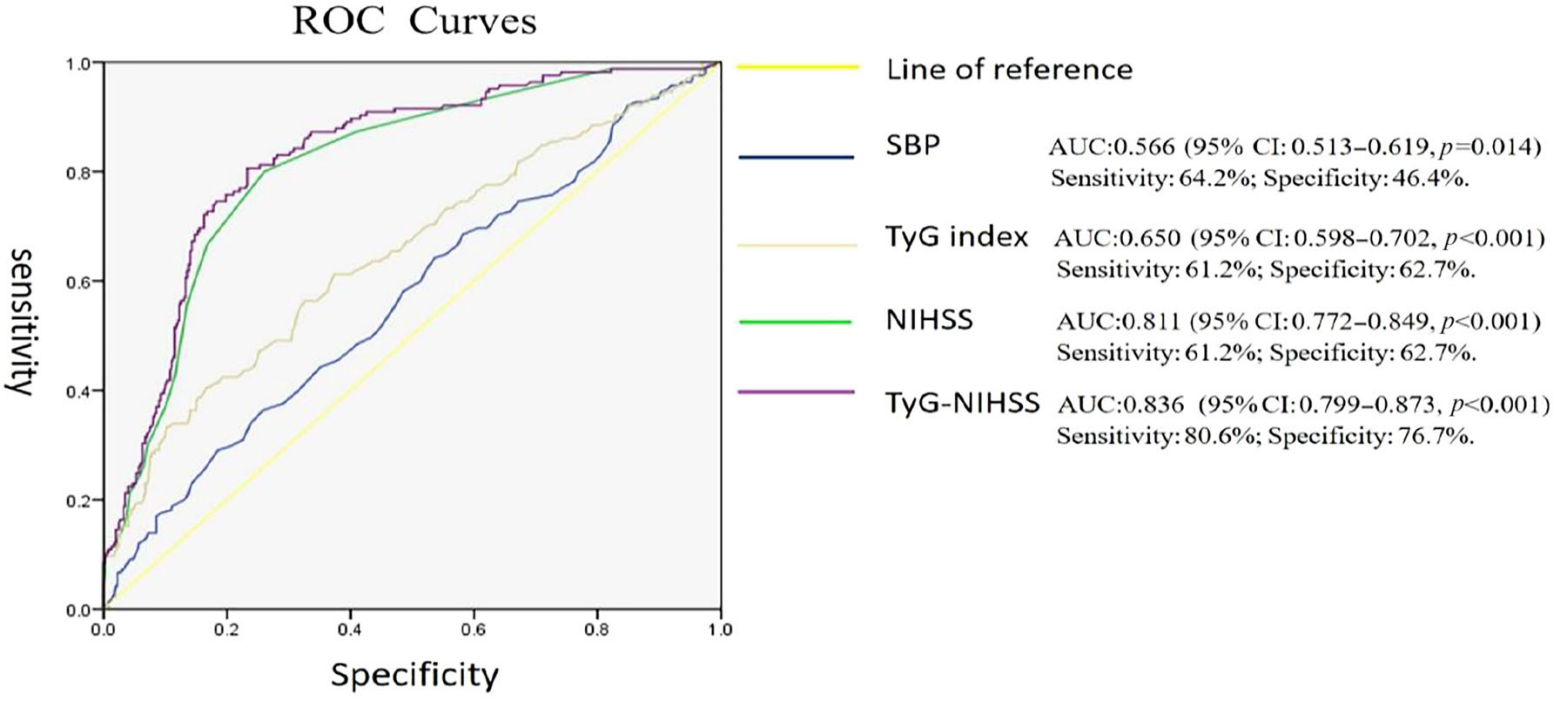
Predictive ability of independent risk factors for 3‐month poor functional prognosis. AUC, area under the curve; NIHSS, National Institutes of Health Stroke Scale; ROC Curves, receiver operating characteristic curve; SBP, systolic blood pressure; TyG index, triglyceride–glucose index; TyG‐NIHSS, The triglyceride–glucose index combined with the admission NIHSS score.

ROC curve analysis showed that the AUC for the TyG index to predict poor functional prognosis at 3 months in AIS patients was 0.650 (95% CI: 0.598–0.702, *p* < 0.001), with the optimal cut‐off value of 8.905, a sensitivity of 61.2%, and a specificity of 62.7%. This result indicates that when the TyG index is used alone as a predictor, AIS patients with a TyG index higher than 8.905 have a significantly increased risk of 3‐month poor functional prognosis. The AUC for the TyG‐NIHSS to predict poor functional prognosis at 3 months in AIS patients was 0.836 (95% CI: 0.799–0.873, *p* < 0.001), with the optimal cutoff value of 0.281, a sensitivity of 80.6%, and a specificity of 76.7%. This result indicates that when using TyG‐NIHSS as a predictor, AIS patients with parameters above 0.281 have a significantly increased risk of 3‐month poor functional prognosis (Figure 1).

## 4 Discussion

Stroke is currently a leading cause of death and disability worldwide. Therefore, accurate prognostic assessment of stroke patients is of paramount importance. This study analyzed clinical data and laboratory parameters of 564 AIS patients and found that the TyG index in the 3‐month poor functional prognosis group was significantly higher than that in the good functional prognosis group, with a statistically significant difference between the two groups (*p* < 0.05). Further exploration through logistic regression modeling revealed that admission TyG index was an independent risk factor for early poor functional prognosis in AIS patients. It demonstrated moderate predictive value, with an AUC of 0.650 (95% CI: 0.598–0.702, *p* < 0.001), predictive sensitivity of 61.2%, and specificity of 62.7%. These findings are generally consistent with previous research on the TyG index for early functional prognosis in stroke. However, our results indicate that although the TyG index is significantly associated with 3‐month poor functional prognosis, its predictive ability as an independent predictor is limited. This may be attributed to the fact that early stroke functional outcomes are influenced by multiple factors, and the TyG index, as a surrogate marker for IR, represents an important but not decisive aspect of stroke pathophysiology. Despite its limited efficacy as an independent predictor, the TyG index has the advantage of being easily obtainable and cost‐effective, making it more operationally feasible in clinical practice. A retrospective study involving 3216 AIS patients from 22 hospitals found that higher TyG index levels were associated with poorer functional outcomes at discharge and higher in‐hospital mortality rates, suggesting that the TyG index is significantly correlated with early prognosis in AIS patients (Miao et al. 2023). Another multicenter prospective study found that higher TyG index levels predicted worse 90‐day functional outcomes in ischemic stroke patients receiving intravenous thrombolysis (Lin et al. 2022). These research findings provide strong evidence supporting the TyG index as an early functional prognostic factor for AIS.

The TyG index, as a reliable surrogate marker for IR, influences stroke prognosis through mechanisms involving hyperglycemia, hyperlipidemia, and hypertension, which are established risk factors for stroke (Lu et al. 2024; Jahdkaran and Sistanizad 2025; Guo et al. 2025). First, the TyG index directly reflects the combined effects of glucose and triglycerides on stroke outcomes. Hyperglycemia creates a vicious cycle of “hyperglycemia‐insulin resistance,” ultimately leading to severe neurological damage (Nagayach et al. 2024; Yki‐Jarvinen 1992; Shannon et al. 2018; Hoy et al. 2007). Hypertriglyceridemia induces oxidative stress and thrombosis, resulting in early neurological deterioration in stroke patients (Deng et al. 2019; Bakker et al. 2000). Second, in patients with concurrent IR and hypertriglyceridemia, these conditions mutually exacerbate each other, further aggravating the degree of IR (Mikhail 2009; Eckel et al. 2005). This promotes cytokine release and apoptosis of vascular smooth muscle cells, macrophages, and endothelial cells, thereby worsening brain injury (Kim et al. 2020; Lee et al. 2024; Wei et al. 2023). Third, IR can enhance sympathetic nervous activity and catabolism in muscles, leading to decreased limb muscle mass and directly affecting patients' motor function (Sosa et al. 2025). In summary, the poor stroke prognosis associated with the TyG index results from the combined effects of multiple pathophysiological factors, including IR, hyperglycemia, and hypertriglyceridemia.

Due to the limited predictive ability of the TyG index alone, we further constructed a combined logistic regression model incorporating the TyG index and admission NIHSS score as two independent predictors, yielding a TyG‐NIHSS model with an AUC of 0.836 (95% CI: 0.799–0.873, *p* < 0.001), sensitivity of 80.6%, and specificity of 76.7%. The results demonstrate that TyG‐NIHSS possesses good predictive ability, exceeding the traditionally recognized threshold for clinical significance and markedly higher than the predictive capacity of the TyG index alone in our study. This indicates that the TyG‐NIHSS model exhibits strong predictive capability and high predictive accuracy. The explanation for this result lies in the fact that the admission NIHSS score in the TyG‐NIHSS model reflects the severity of acute brain injury at admission (Kazi et al. 2021), while the TyG index reveals the metabolic mechanisms underlying poor outcomes in ischemic stroke (Li et al. 2023; Sojitra et al. 2023). The combined indicator simultaneously encompasses both the severity of brain injury and the metabolic pathophysiological processes in stroke patients. The model integrates risk factors from different dimensions, thereby providing more comprehensive and accurate predictive capability.

Currently, most research on the relationship between TyG index and stroke has focused on stroke incidence, recurrence rates, and mortality risk, with relatively limited studies on early functional prognosis (Nayak et al. 2024). Our work enriches the research on the relationship between the TyG index and clinical stroke outcomes, and our findings support the hypothesis that an elevated TyG index is associated with poor functional outcomes. Additionally, our study successfully developed and validated the TyG‐NIHSS combined prediction model. This model demonstrates good predictive ability for 3‐month early functional prognosis in AIS patients.

Certainly, our study has several limitations. First, this is a retrospective study with a limited sample size. The results still require further validation through multicenter, prospective experimental studies. Second, all study subjects were Chinese residents, and we cannot determine whether our findings can be extrapolated to other regions and populations worldwide. Third, the study did not exclude factors such as the quality of acute‐phase rehabilitation, previous secondary prevention strategies, CYP2C19 polymorphisms, and the degree of family support, which would better elucidate the relationship between the TyG index alone and the TyG‐NIHSS combined parameter with stroke prognosis. In the next step, we will expand the sample size and conduct more specific risk stratification for the TyG‐NIHSS combined prediction model to enable early identification of high‐risk stroke patients for closer monitoring and more aggressive treatment.

## 5 Conclusion

This retrospective study aimed to investigate the relationship between the TyG index and clinical outcomes in ischemic stroke patients. Our findings demonstrate that the TyG index has a significant correlation with clinical outcomes. The TyG index is an independent risk factor for 3‐month poor functional prognosis in AIS patients, exhibiting moderate predictive ability. Specifically, higher admission TyG index levels are associated with increased risk of poor functional outcomes at 3 months. However, TyG‐NIHSS represents a highly discriminative multivariate model. This model demonstrates good predictive ability, significantly superior to that of the TyG index alone in our study. This indicates that the TyG‐NIHSS model possesses strong predictive capability and high predictive accuracy.

## Author Contributions

Zhuqing Wu contributed to the design and implementation of the study, the statistical analysis of the data, and the writing of the paper. Yueyu Zhang, Qiuwan Liu, Kangrui Zhang, and Xiaoqiang Wang contributed to data collection and statistical analysis. Juan Shi and Chi Zhang contributed to data collection and implementing the research. Juncang Wu contributed to the design of the study, and the direction and revision of the paper. All authors have read and agreed to the published version of the manuscript.

We thank all the researchers and staff of the National Advanced Stroke Center at Hefei Second People's Hospital for their help. Thanks to Professor Juncang Wu for the guidance of this study.

## Funding

This work was supported by Hefei Key Common Technology Research and Development Project (GJ2022SM07).

## Ethics Statement

This study was approved by the medical ethics committee of the Second People's Hospital of Hefei, and all participants or their legal representatives signed informed consent forms.

## Consent

The authors have nothing to report.

## Conflicts of Interest

The authors declare no conflicts of interest.

## Peer Review

The peer review history for this article is available at https://publons.com/publon/10.1002/brb3.71013.

## Data Availability

The datasets generated for this study are available on request to the corresponding author.
